# Tree-level almond yield estimation from high resolution aerial imagery with convolutional neural network

**DOI:** 10.3389/fpls.2023.1070699

**Published:** 2023-02-15

**Authors:** Minmeng Tang, Dennis Lee Sadowski, Chen Peng, Stavros G. Vougioukas, Brandon Klever, Sat Darshan S. Khalsa, Patrick H. Brown, Yufang Jin

**Affiliations:** ^1^ Department of Land, Air, and Water Resources, University of California, Davis, Davis, CA, United States; ^2^ Department of Biological and Agricultural Engineering, University of California, Davis, Davis, CA, United States; ^3^ Department of Plant Sciences, University of California, Davis, Davis, CA, United States

**Keywords:** CNN, deep learning, yield prediction, multispectral imagery, almond, UAV/drone

## Abstract

**Introduction:**

Estimating and understanding the yield variability within an individual field is critical for precision agriculture resource management of high value tree crops. Recent advancements in sensor technologies and machine learning make it possible to monitor orchards at very high spatial resolution and estimate yield at individual tree level.

**Methods:**

This study evaluates the potential of utilizing deep learning methods to predict tree-level almond yield with multi-spectral imagery. We focused on an almond orchard with the ‘Independence’ cultivar in California, where individual tree harvesting and yield monitoring was conducted for ~2,000 trees and summer aerial imagery at 30cm was acquired for four spectral bands in 2021. We developed a Convolutional Neural Network (CNN) model with a spatial attention module to take the multi-spectral reflectance imagery directly for almond fresh weight estimation at the tree level.

**Results:**

The deep learning model was shown to predict the tree level yield very well, with a R2 of 0.96 (±0.002) and Normalized Root Mean Square Error (NRMSE) of 6.6% (±0.2%), based on 5-fold cross validation. The CNN estimation captured well the patterns of yield variation between orchard rows, along the transects, and from tree to tree, when compared to the harvest data. The reflectance at the red edge band was found to play the most important role in the CNN yield estimation.

**Discussion:**

This study demonstrates the significant improvement of deep learning over traditional linear regression and machine learning methods for accurate and robust tree level yield estimation, highlighting the potential for data-driven site-specific resource management to ensure agriculture sustainability.

## 1 Introduction

Over 2.2 million ha of land produces about 4.1 million metric tons of almonds in 2020 globally, with United States (US) as the largest producer (FAO, 2022). About 80 percent of the world’s almonds are produced in California’s irrigated land, generating about $5bn “farm gate value” and an additional $3 billion of indirect and induced values (CDFA, 2022). In the last two decades, the total acreage of almond orchards in California doubled and became the state’s second largest agricultural commodity. The continued expansion of water and fertilizer-intensive tree crops, coupled with climate change, poses a threat to the long-term sustainability of almond industry, despite ongoing research and outreach efforts focused on tree crops (Khalsa et al., 2022). Excessive groundwater pumping especially during drought years, for example, has caused a significant drop of aquifer’s water depths in Central Valley (Fulton et al., 2019). Groundwater has also been degraded due to nitrogen leaching from agricultural fields (Harter, 2009). One out of ten public water supply wells in California have nitrate levels exceeding the maximum contamination level (Harter, 2009).

In response to these challenges, various regulatory programs have been implemented in California over the past decade, requiring growers to increase the efficiency of irrigation and nitrogen use (Rudnick et al., 2021). Meeting these regulations will require more precise and adaptive irrigation and nitrogen management strategies. In particular, a change from whole-field management to zonal and even tree-specific precision agricultural practices is critical for maximizing ‘crop per drop or lb of N’, considering large yield variability within an individual almond orchard (Jin et al., 2020). Accurate yield estimation and prediction is a missing link in current nitrogen management tool, although the guidance is available on N fertilization given the expected almond yield for a particular orchard. An improved understanding of within-field yield variability is also needed for adaptive on-farm management to close the yield gap (Jin et al., 2020). Reliable yield estimation can also help with insurance and market decisions, which rely on the understanding of mean and variability of yields at the field scale (Lobell et al., 2015).

Both mechanistic simulation models and statistical approaches have been used for yield estimation (Hodges et al., 1987; Dzotsi et al., 2013; Burke and Lobell, 2017; Kang and Özdoğan, 2019; Sidike et al., 2019). The process models simulate crop growth, nutrient cycling, soil-plant dynamics, and energy and water balance under various climate and management scenarios (Zhang et al., 2019; Archontoulis et al., 2020), such as the Agricultural Production Systems Simulator (APSIM) model (Keating et al., 2003). Although powerful, it is challenging to calibrate these models across different sites, because of the complexity of the biological processes (Jagtap and Jones, 2002). These models often require extensive biotic and abiotic data as input, such as soil properties, which may not be available at the field or finer scale (Sakamoto et al., 2013; Zhang et al., 2019). Moreover, the majority of crop models focus on row crops such as corn, soybean, barley, and etc., while the simulation of tree crops with complicated physiological processes is very limited (Keating et al., 2003).

Statistical models, on the other hand, are based on the empirical relationships learned from the observed yield data and the factors affecting production, instead of simulating complex biophysical processes (Medar and Rajpurohit, 2014). Regression models, for example, have been developed to quantify the impact of climate on agriculture production at county and state level (Lobell et al., 2007; Lobell and Field, 2011; Mourtzinis et al., 2015; Xu et al., 2016). Studies have shown that the recent climatic trends have mixed effects on tree crop yields in California (Lobell et al., 2007; Lobell and Field, 2011). Across the US, it has been estimated that warming will lead to reduction in soybean and maize production in the Midwest (Mourtzinis et al., 2015; Xu et al., 2016). All these statistical studies provide guidance for county, state or nation-wide climate mitigation and adaptation strategies. However, the utility of these coarse scale empirical models is limited in terms of informing growers for their on-farm resource management for individual fields or trees.

Recent advancement of remote sensing technologies enables plant monitoring across a range of spatial and temporal resolutions, opening doors for data-driven yield estimation at the field scale (Shahhosseini et al., 2020; van Klompenburg et al., 2020; Rashid et al., 2021; Muruganantham et al., 2022). Both traditional and machine learning methods have been developed to relate field surveyed yield data with remote sensing metrics and other environmental drivers (Burke and Lobell, 2017; Lambert et al., 2018; Hunt et al., 2019; Zhang et al., 2019). Burke and Lobell (2017) found that the linear regression model, driven by vegetation indexes (VIs) derived from high resolution multi-spectral images from Terra Bella satellite at 1m, predicted well the yield for maize fields in west Kenya. Machine learning models such as random forest and gradient boosting trees have also been developed to predict yield for individual fields over almond tree crops by integrating Landsat VIs and weather data in California (Zhang et al., 2019), over wheat in United Kingdom using Sentinel-2 VIs (Hunt et al., 2019), and over cotton, maize, millet and sorghum in Mali using Sentinel-2 VIs (Lambert et al., 2018).

Most recently more complex deep learning models such as Deep Neural Network, Convolutional Neural Network (CNN), and Recurrent Neural Network have been introduced to improve yield estimation with large remote sensing datasets, due to their improved performance over traditional statistical approaches (Ball et al., 2017; You et al., 2017; Cai et al., 2018; Kang and Özdoğan, 2019; Khaki and Wang, 2019; Sidike et al., 2019; Kang et al., 2020; Khaki et al., 2020; Ma et al., 2021). The Bayesian neural network model, for example, has been shown to predict county-level corn yield well in twelve Midwestern states of US (R^2^ = 0.77), using VI time series from MODIS imagery, climate variables, soil properties, and historical average yield (Ma et al., 2021). A limited studies applied recurrent neural network framework such as Long Short Term Memory models to take into account of sequential imagery and weather for county-level corn yield in combination with CNN; their models outperform the traditional regression and machine learning models (You et al., 2017; Khaki et al., 2020). Shahhosseini et al. (2021) also explored a hybrid approach to integrate features from crop modeling to machine learning models and found the importance of hydrological inputs for yield estimation in the US corn belt. At field scales, data assimilation technique has been explored to incorporate the remote sensing observations of canopy development into the Decision Support System for Agrotechnology Transfer (DSSAT) crop model for corn yield mapping over the US corn belt (Kang and Özdoğan, 2019). However, most of the studies still use human-engineered index-based feature extraction method, such as some widely used vegetation index and contextual information derived from imagery, to predict yield and do not explore the potential of learning-based feature extraction with deep learning models that directly use multi-spectral imagery as input.

In order to capture variations of crop yield among individual plants for precision management, higher spatial resolution observations of canopy structure and conditions are required, such as those from very high-resolution commercial satellite and aerial imagery (Sidike et al., 2019; Maimaitijiang et al., 2020). Recent advances in computer vision and deep learning technology further unlock the power of centimeter imagery for fine scale yield estimation at individual plant or sub-field level. Chen et al. (2019) developed a region-based CNN model to detect and count the number of flowers and strawberries at plant level from the RGB drone imagery and found an overall counting accuracy of 84.1%. Another study integrated multi-spectral and thermal drone imagery with machine learning and deep neural network models to estimate the sub-field soybean yield in US (Maimaitijiang et al., 2020). However, the study on plant-level yield variation is still very limited and the majority focuses on row crops, mostly due to the lack of field-based yield database for individual plants, especially for tree crops.

We here took advantage of a unique individual tree harvesting data and aerial imagery of multiple spectral bands at 30cm spatial resolution over an almond orchard in California’s central valley, to explore the potential of deep learning for tree level almond yield estimation. Specifically, we aimed to address the following questions: (i) how CNN model can be used to estimate almond yield for each individual tree, based on very high resolution multi-spectral imagery; and (ii) what is the capability of the trained CNN models in capturing the within-field almond yield variation; and (iii) what is the relative importance or added value of the observations in the red edge part of the spectrum, a spectral band increasingly available in recent imaging systems, with regard to almond yield estimation.

## 2 Materials

### 2.1 Study orchard and Individual tree harvest data

**Figure 1:**
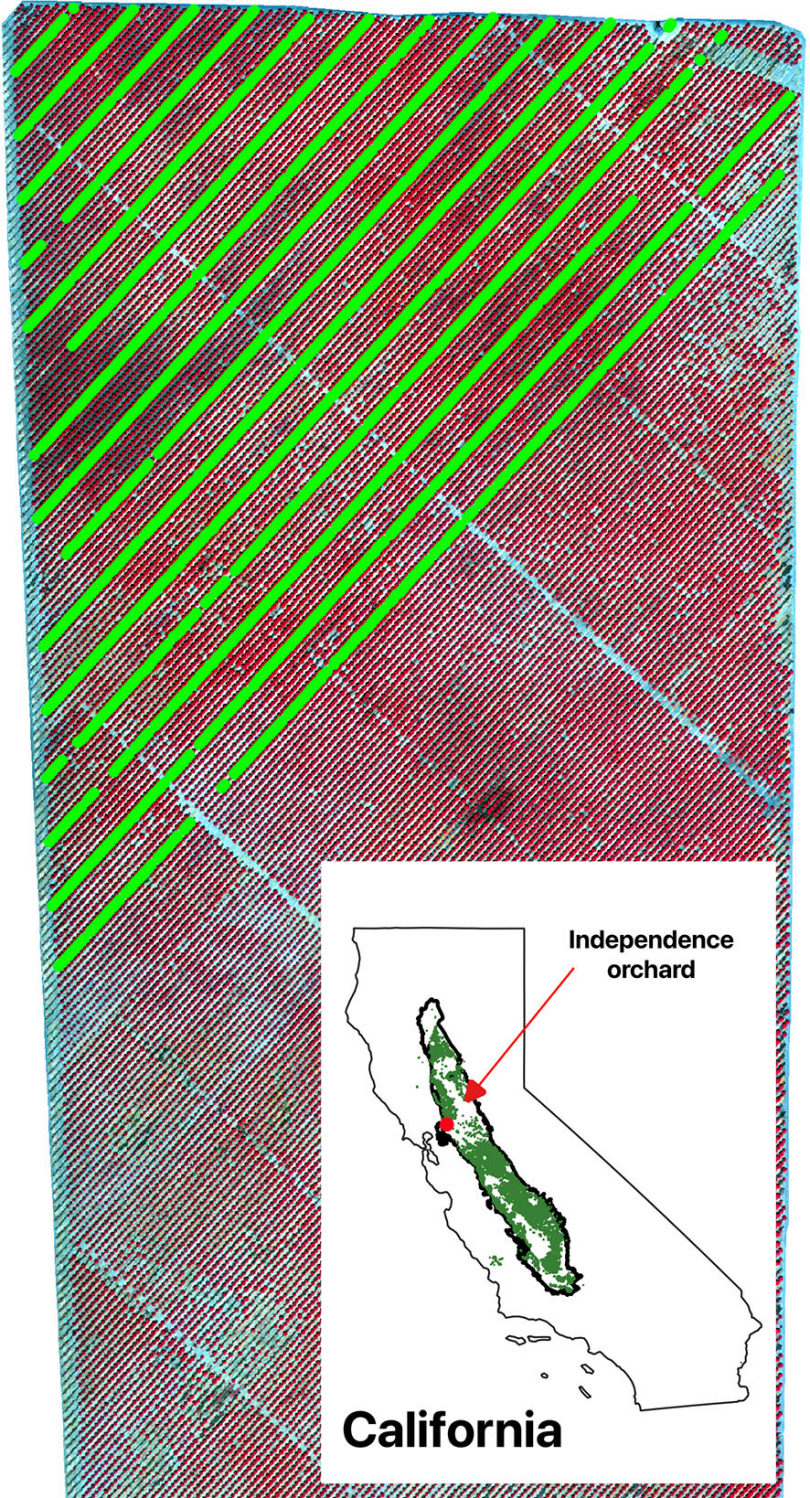
Study orchard as shown by the color infrared composite of CERES aerial imagery acquired on July 29, 2021. Individual trees with yield measurements were shown as green dots. The inset shows the location of the study orchard among all almond orchard fields (green) in California’s Central Valley (black polygon).

This study was conducted over an almond orchard with a size of 2 squared kilometers in Vacaville, California, USA (**Figure 1**). Under a typical Mediterranean climate, the area experiences hot dry summer with average daily max temperature in July of 34 °C and cool winter with average daily minimum temperature in January of 3.7 °C. Mean annual precipitation is 63 ( ± 21) cm and the majority rainfall occurs from November to March (BestPlaces, 2022; Cedar Lake Ventures, 2022; WRCC, 2022). For almond tree, the water usage increases gradually from March to July, and decreases from July to October (Athwal, 2021). The hot and dry summer requires large amount of irrigation water usage to support crop growing, which mainly comes from groundwater and surface water including Lake Berryessa and Putah Creek (SID, 2012; BoR, 2022).

The orchard was planted with a self-fertile productive almond cultivar, ‘Independence’, between 2015 and 2017. Within the orchard, rows are oriented northeast to southwest in parallel with prevailing winds, and the average row spacing is about 6 m and the average spacing between trees along the same row is about 4.5 m. Almond trees bloom between late February and early March, followed by leaf out, fruit set and rapid growth, reaches full canopy typically in June or early-July, and fruit maturity progresses through summer. Almonds are typically harvested from August to October, and trees become dormancy during the winter season.

We designed an automatic weighing system attached to the commercial almond harvester to measure the almond yield of an individual tree (**Figure S1**). The yield (including wet hulls and shells) measurements were made for each individual tree every seven rows in the north-west portion of the orchard between August 23 and August 27 in 2021 (**Figure 1**). A total number of 1,893 trees were individually harvested, with an average fresh weight yield of 53.1 ± 17.6 kg per tree. The location of each sampled trees was also recorded. Large yield variation was found among individual trees with a coefficient of variation of 33.1% and interquartile range of 24.3 kg per tree.

### 2.2 Aerial imagery acquisition and processing

Multi-spectral aerial imagery was acquired on July 29, 2021, about one month ahead of the harvest, by CERES Imaging (Oakland, USA.) A multi-spectral imaging camera was integrated with a crop duster plane flying at 6,000 ft above the ground, resulting in images with a 0.3-meter spatial resolution. Four spectral bands are centered around 800 nm (near infrared), 717 nm (red edge), 671 nm (red), and 550 nm (green), with a spectral resolution of 10 nm (the full width at half maximum). The image was acquired near local solar noon to minimize the shadow effects.

### 2.3 Tree identification and location extraction from imagery

**Figure 2:**
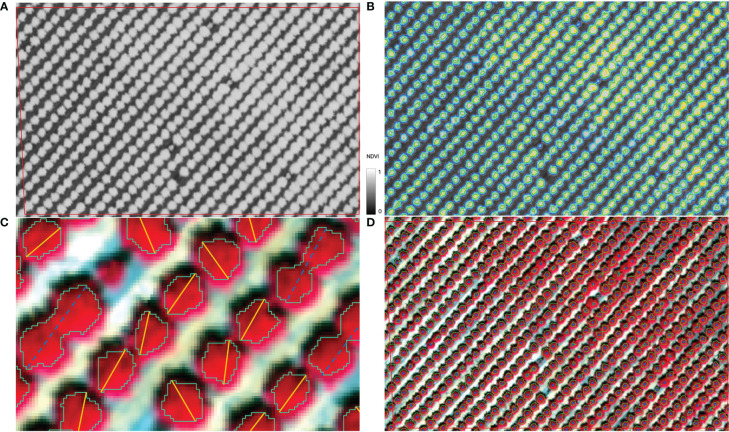
Illustration of individual tree identification workflow: **(A)**. NDVI map from CERES imagery; **(B)**. Segmented tree crowns with various NDVI threshold values, e.g., the blue polygon represents the boundaries from the segmentation with a NDVI threshold of 0.6; **(C)**. For each polygon layer identified using a particular NDVI threshold, remove those crown polygons whose major axis (dashed blue line) were longer than the expected maximum tree crown diameter, roughly the tree planting spacing along the orchard row; **(D)**. Final tree crowns by combining all layers of potential crown polygons and center locations of all individual trees.

For each individual tree, extracting its center location from CERES imagery is needed in order to match the tree yield record from the harvester and to clip the corresponding image block as CNN input. We developed a multi-stage segmentation method to identify all individual crowns with varying canopy sizes, especially over a mature orchard. First, Normalized Difference Vegetation Index (NDVI) was calculated for each pixel from the red and near infrared bands of the CERES aerial imagery (**Figure 2A**). Second, NDVI imagery was segmented based on the NDVI threshold to identify potential tree crowns automatically (**Figure 2B**). Lower NDVI threshold tended to be more inclusive in identifying canopy pixels and resulted in a tree crown boundary with multiple inter-connected trees in it; whereas higher NDVI threshold separated individual tree crowns better but may miss smaller trees (**Figure 2B**). We therefore applied seven NDVI thresholds ranging from 0.60 to 0.83 (**Table S1**), producing seven layers of potential tree crown polygon maps. Third, for each layer, those polygons that actually had multiple trees were removed, based on the comparison of the polygon major axis length and the orchard tree spacing (**Figure 2C**). The assumption is that one single tree crown diameter can’t exceed the spacing between adjacent trees. Finally, by taking advantage of higher threshold’s capability of separating individual trees and lower NDVI threshold’s capability of identifying small trees, we combined those seven potential single tree crown polygons iteratively, based on their spatial relationships, into one final tree crown boundary optimal for tree center extraction. The goal was to remove the redundancy among those layers yet maintain the largest crown size. Starting from the crown polygons (smallest size), typically associated with higher NDVI threshold value, if it was spatially within the crown polygon (larger) identified by the lower threshold value, it was deleted; otherwise, it was added to the final single tree crown polygons map. By iterating this step, we created a final version of single tree crown polygons map (**Figure 2D**). Finally, the tree locations were extracted from the centroid coordinates of all the segmented tree crown polygons.

For quality control, the extracted tree locations were plotted over the CERES imagery for visual examination. For example, those trees with very small or large crowns were carefully examined against CERES imagery to ensure the location accuracy. To further ensure the alignment with the locations of the individually harvested trees, a visual check of the locations of starting, ending, and some randomly selected trees within the harvested rows was also conducted. All these processes were done in Python and QGIS.

## 3 Methods

### 3.1 Convolutional neural network architecture

The Convolutional neural network (CNN), a most established deep learning algorithm, is developed to estimate fresh almond yield with multi-spectral aerial images as inputs. CNN has a unique ability to automatically and adaptively learn spatial hierarchies of important features that summarize the presence of detected features in the input image for a particular predictive modeling problem (LeCun et al., 2015). The extreme efficiency in dimensionality reduction of the CNN model makes it unnecessary to conduct any feature extraction work, which increases computation efficiency and improves estimation accuracy. A surge of interest in CNN deep learning has emerged in recent years due to its superior performance in various fields (Lobell et al., 2015; Yamashita et al., 2018; Kattenborn et al., 2021; Li et al., 2021).

A CNN is typically composed of a stacking of three types of layers, i.e., convolution, pooling, and fully connected layers (LeCun et al., 2015). The first two perform feature extraction, whereas the third maps the extracted features into final output, such as yield. As a fundamental component of the CNN architecture, a convolutional layer typically consists of a combination of linear and nonlinear operations, i.e., convolution operation and activation function. A convolution is a simple application of a spatial filter (or kernel) to an input image that results in an activation. Repeated application of the same filter to an input result in a map of activations called a feature map. A small grid of parameters called kernel, an optimizable feature extractor, is applied at each image position, which makes CNNs highly efficient for image processing. The kernel values are optimized during the model training process to extract features from input data based on the model’s task. The outputs of a linear operation such as convolution are then passed through a nonlinear activation function, e.g., the most commonly used rectified linear unit (ReLU). Batch normalization can also be applied as an optimization strategy to increase the model training efficiency, although it is not a solid requirement of the CNN model. To reduce the dimensionality of the extracted feature maps, a pooling layer provides a down-sampling operation by aggregating the adjacent values with a selected aggregation function, such as taking maximum value within the predefined window size. Similar to convolution operations, hyperparameters including filter size, stride, and padding are set in pooling operations. As one layer feeds its output into the next layer, extracted features can hierarchically and progressively become more complex.

To improve CNN model’s overall performance, the spatial attention module is recently introduced into the CNN architecture by combining a global average pooling layer and the following dense layers (Woo et al., 2018; Sun et al., 2022; Zhang et al., 2022). Global average pooling layer is usually applied once to downscale the feature maps into 1-D array by averaging all the elements in each feature map, while retaining the depth of the feature maps. Dense layer then connects the final feature maps to the final output of the model with learnable weights *via* model training. The combination of a global average pooling layer and the following dense layers helps the CNN model focus more on the relevant features and thus improves.

### 3.2 CNN configuration and optimization

**Figure 3:**
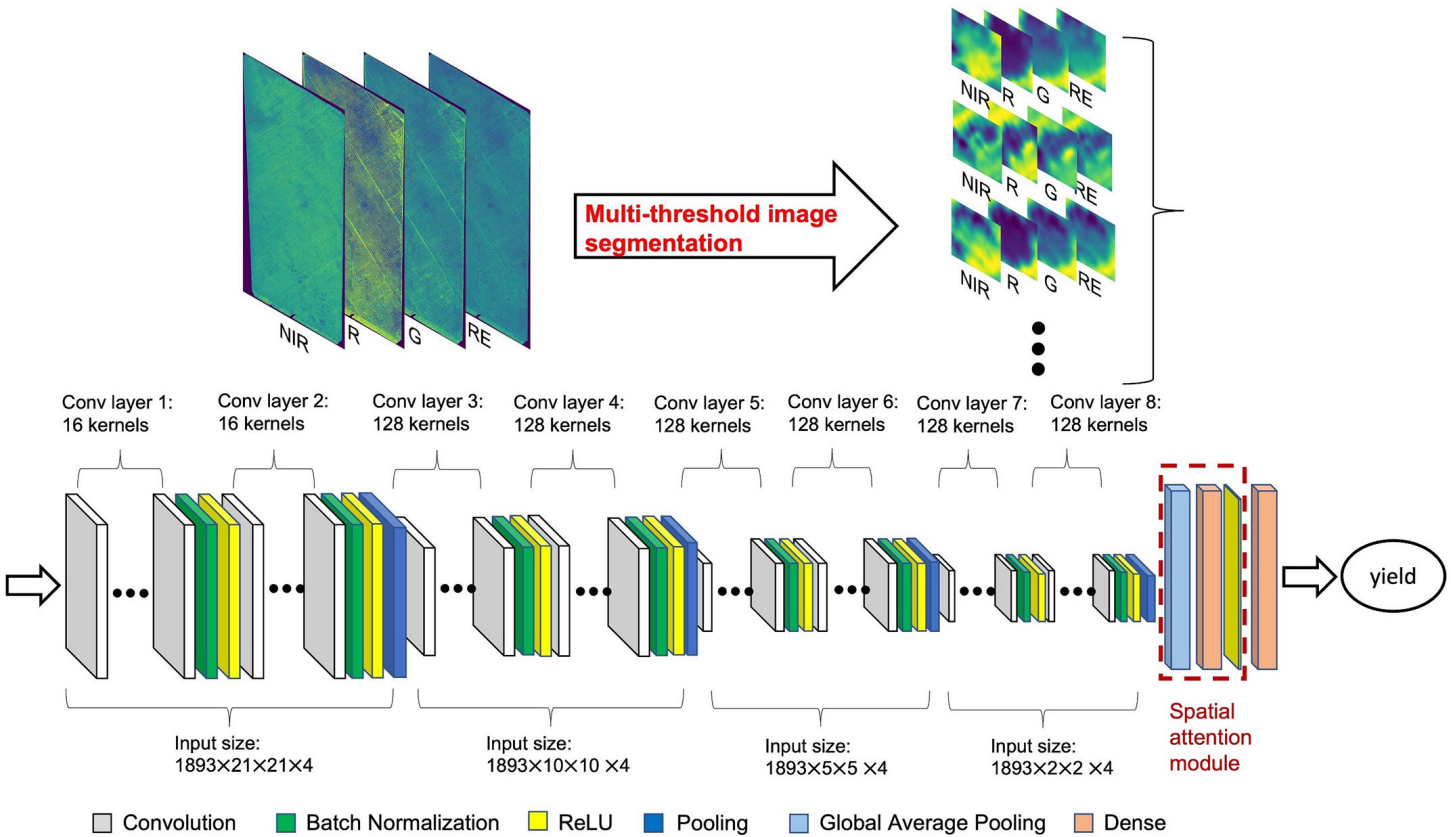
CNN model structure for tree-level yield estimation with multi-spectral aerial imagery. Input size represents total number of tree samples × image block height × image block width × number of bands. For each tree sample, an image block was clipped for each one of the four band imagery, with 21 by 21 pixels (at a 30cm resolution) centered at the identified tree center location.

TensorFlow (Abadi et al., 2016), Keras (Chollet, 2015), and KerasTuner (O’Malley et al., 2019) libraries in Python were used for CNN model tuning and training processes. The CNN model took the image blocks, centered around each individual almond tree crown, from CERES images at 0.3 m resolution, for 4 reflectance bands (R, G, NIR, and RE) as inputs to estimate the individual tree almond yield (**Figure 3**). We started with the minimum block size of 21 × 21 pixels, equivalent to a 3m radius centered around each tree crown center and thus representing areas slightly bigger than one tree crown size. For each tree sample, we first identified the corresponding CERES pixel containing the tree center (as described in Section 2.3 location), and then clipped an image block extending 10 pixels towards all four directions from the center, for each band. This step resulted in 21 × 21 × 4 multi-spectral imagery associated with each individual tree crown as the input to the CNN model.

The CNN model training process is to find kernels in the convolutional layers and weights in the dense layers to minimize the differences between model estimations and ground measurements on a training dataset. The Mean Squared Error (MSE) loss function was applied for the CNN model training, which calculates the average of the squared differences between model estimations and actual values. To efficiently optimize the kernels and weights within the CNN model, the Adam optimization algorithm (Kingma and Ba, 2014) is used, which extends the stochastic gradient descent algorithm by calculating individual learning rates for different parameters based on the estimates of first and second moments of gradients. 5-fold cross validation (CV) is applied to randomly split the data into separate training and testing sets. The overall model performance is evaluated based on the average performance over the testing set in each fold. The Bayesian optimization algorithm is developed to select the CNN hyper-parameters automatically.

The general setup of the possible CNN structures for the Bayesian optimization algorithm are as follows: three to four convolutional blocks followed by a spatial attention module with a global average pooling layer and two fully connected dense layers. For the first dense layer, there are 30 to 100 neurons followed by a dropout layer. For each convolutional block, there are 16 to 128 convolutional layers (kernels) followed by a batch normalization and pooling layers, then another 16 to 128 convolutional layers followed by a batch normalization, pooling and ReLU activation layers. The pooling layers in each convolutional block can be either average pooling or max pooling. The overall architecture of the CNN model for the Bayesian optimization algorithm is shown in **Figure S3**. For model compiler, the Bayesian optimization algorithm selects learning rate varying from 10^-4^ to 10^-2^ with Adam optimizer. For the Bayesian optimization algorithm itself, the maximum trail number was set to 50, and for each trail, the batch size is 128 with 100 epochs.

To investigate the impact of input image block size used for the CNN model and explore how the neighboring trees potentially influence yield estimation, another two separate CNN models were built with an input image size of 41 × 41 pixels (roughly 6m radius) and 61 × 61 pixels (9m radius), respectively. To understand the contribution of the red edge band to the yield estimation, a reduced CNN model was constructed by excluding red edge reflectance as input, hereafter called “reduced CNN model”, considering that red edge band is not as widely used for aerial imaging as the other three bands. Similarly, another 14 sets of reduced CNN models were further built with all the combinations of different reflectance bands as input and compared how they influenced model’s yield estimation accuracy (**Table S2**).

### 3.3 Traditional machine learning model estimations

For comparison purposes, Other statistical models were also built for individual tree level almond yield estimation, including stepwise linear regression as a baseline for linear relationships and four traditional machine learning approaches. The Scikit-learn (Buitinck et al., 2013) and hyperopt (Bergstra et al., 2013) libraries were used for building support vector regressor (SVR) (Platt, 1999), random forest (RF) (Breiman, 2001), and extreme gradient boosting (XGB) models (Chen and Guestrin, 2016). Additionally, a DNN model was also developed using the same libraries as CNN model. The traditional machine learning models use the human-engineered index-based feature extraction method to predict almond yield, which differs from the CNN model that directly takes imagery as input. By comparing traditional machine learning models against CNN model, it helps to evaluate the advantages of applying learning-based feature extraction in yield prediction.

Regression models were built using features at individual tree level as inputs, including VIs and texture. 13 commonly used vegetation indices (VIs) were calculated from CERES multi-spectral imagery, including those sensitive to structure, greenness, and chlorophyll content (as described and summarized in **Table S3** in the supplementary material). A circular buffer with a 2.5-meter radius was used to calculate the zonal statistics of remote sensing metrics, since most tree crowns have diameters less than 5 meters. Tree crown pixels were identified with NDVI greater than 0.5, and the fractional coverage of tree crown within the buffer area was then calculated to represent the size of crown. The average of VI values over the identified crown pixels within the buffer area were also derived to represent the overall biomass of an individual tree. In total, 14 variables were calculated including 13 VIs and one fractional coverage variable.

To extract textural features for each of the four band images, the gray level co-occurrence matrix (GLCM) (Haralick et al., 1973) was applied. The GLCMs were constructed with a moving distance of one pixel and four moving directions. Eight texture measures were calculated from reflectance imagery with a 2x2 moving window, including contrast, dissimilarity, homogeneity, angular second moment, correlation, mean, variance, and entropy (Nichol and Sarker, 2011; Wood et al., 2012). For each individual tree, the corresponding texture features were extracted and averaged from textural images, resulting in a total of 32 texture features.

### 3.4 Accuracy assessment and yield variability analysis

To evaluate models’ performance in predicting almond yield, the predicted and observed individual tree yield from the reserved testing samples were compared, and the coefficient of determination (R^2^), Root Mean Squared Error (RMSE), and RMSE normalized by averaged yield measurement (NRMSE) were calculated. Statistics of these metrics were reported based on 5-fold cross validation.

**Figure 4:**
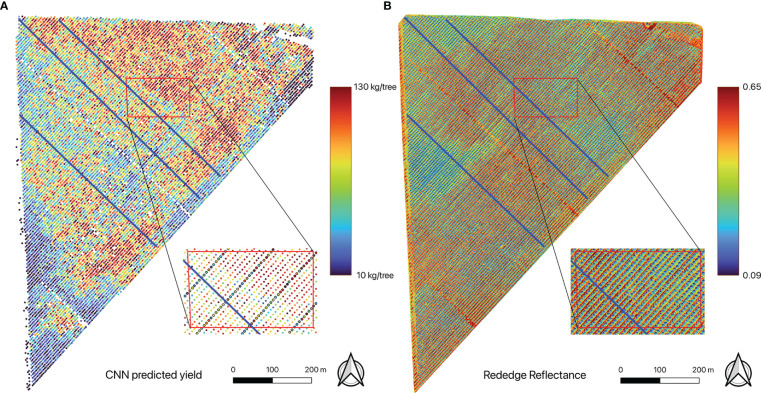
Maps of **(A)** individual tree yield estimated by the CNN mode, showing within-field yield variation, and **(B)** red edge reflectance. A close-up of the yield map shows detailed spatial distribution of the estimated tree yield (those trees with ground yield measurement were indicated by black circles). Also shown are three transects (blue lines) for detailed tree-to-tree yield variation analysis.

For the model with highest accuracy, its capability to capture the within-field yield variations, such as overall spatial patterns, row to row variations, and tree to tree variations along selected transects was also evaluated. For all harvested rows, the yield distribution for all trees within each individual row was analyzed based on CNN estimations. Furthermore, three transects that are perpendicular to the row orientation of the orchard were randomly selected to examine the inter-row yield variations. The locations of the selected transects are shown in **Figure 4** highlighted in blue lines.

## 4 Results

### 4.1 Optimized CNN model and performance

After 50 iterations of Bayesian optimization process during model training, the final optimized CNN model had eight convolutional layers, each of which was followed by a batch normalization and an ReLU activation function. Four max pooling layers were deployed after every two convolutional layers to extract spatial features and reduce image dimension. A global average pooling layer further flatten the image into one-dimension array. A 100-neuron dense layer is introduced. The final one neural dense layer further reduces the input data into a single output value, which directly connects to the tree level yield data (**Figure 3**).

**Figure 5:**
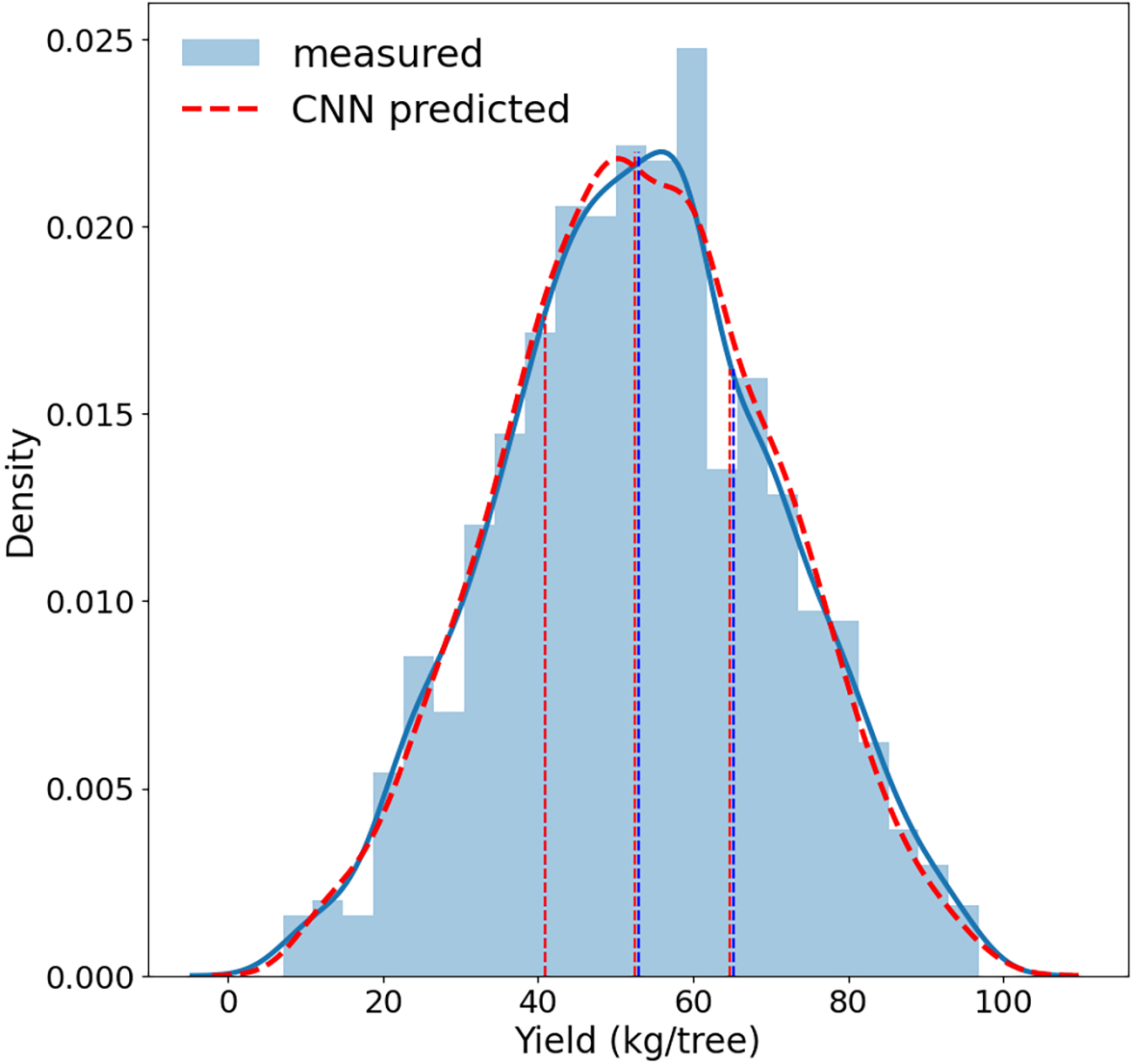
Distributions of almond tree yield predicted by the full CNN model (red) vs. measured by individual tree harvester (blue). Dashed vertical lines represents the 25th percentile, median, and 75th percentile respectively.

**Figure 6:**
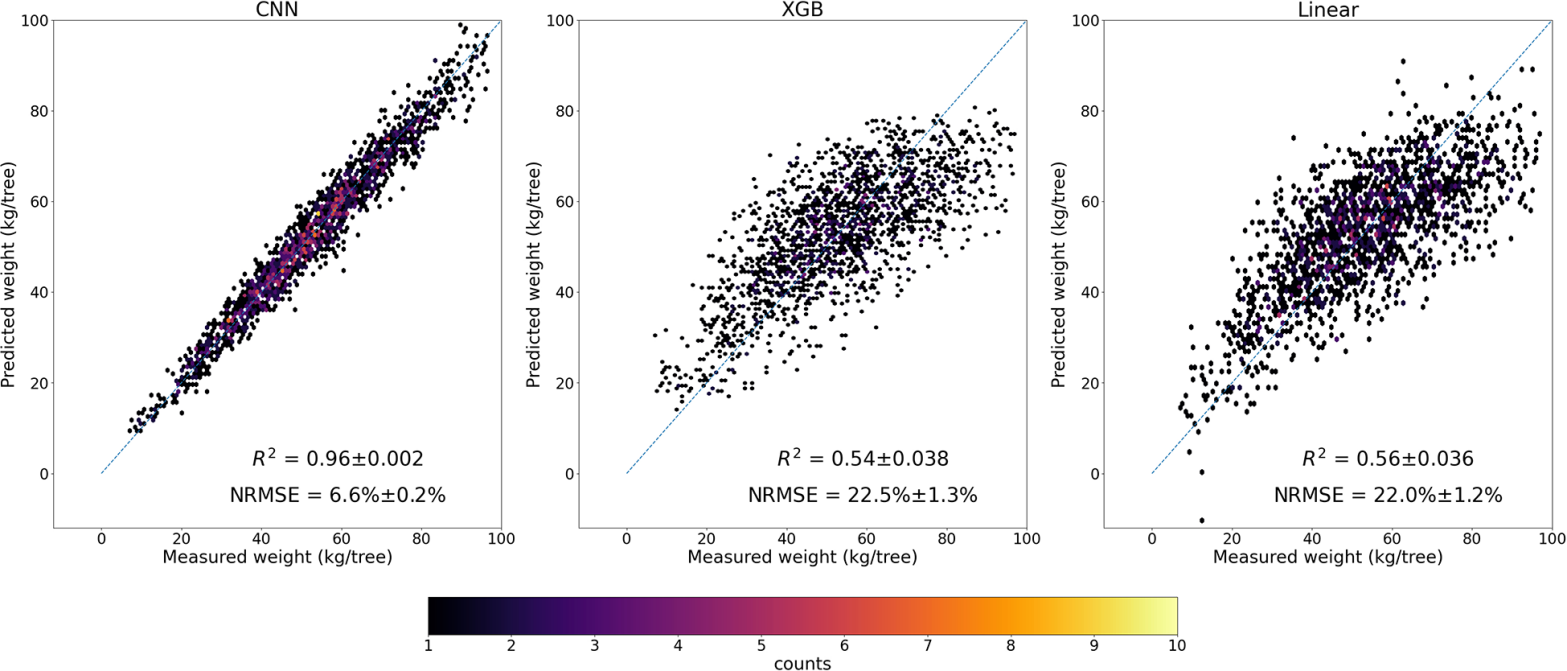
Scatter plots of predicted yield by the full CNN model, XGB, and Linear models vs. measured yield.

The trained CNN full model, with four spectral band imagery as inputs, performed very well in predicting almond yield at the individual tree level. The 5-fold cross validation with the testing data showed that it captured 96% ( ± 0.2%) of tree-to-tree variation in almond yield, with a RMSE of 3.5 kg/tree ( ± 0.11) and a normalized RMSE of 6.60% ( ± 0.2%) (**Figure 5**). The scatter plot of predicted vs. observed tree yield also showed a good agreement (**Figure 6**). The predicted yield by the full CNN model for all individually harvested trees followed very similar distribution as shown by the measurements (**Figure 5**), with a mean yield of 52.9 ± 17.2 vs. 53.1 ± 17.6 kg/tree and the interquartile ranges of 23.8 vs. 24.3 kg/tree. No statistically significant difference was found between predicted and observed tree yield based on the two-tailed t-test (p-value of 0.75).

The performance of the full CNN models with all four bands varied, very slightly, with the size of input image blocks (**Table 1**). For example. when using image blocks covering nine tree crowns, the re-trained CNN model captured 97% of yield variability and had slightly larger uncertainty with a NRMSE 5.2%. However, the estimation bias is larger for CNN models with image blocks covering more tree crowns. Hereafter only the results from the CNN model with 21 × 21 pixels image block size was reported.

**Table 1:** Performance of CNN models with different image block sizes of the input aerial image clipped around each individual tree crown center.

Image block size	Test R^2^	RMSE (kg/tree)	NRMSE	IQR (kg/tree)	Bias (kg/tree)
21×21 pixels	0.96 ( ± 0.002)	3.50 ( ± 0.11)	6.6% ( ± 0.2%)	23.82	-0.181
41×41 pixels	0.95 ( ± 0.017)	4.02 ( ± 0.53)	7.6% ( ± 1.0%)	23.55	1.46
61×61 pixels	0.97 ( ± 0.005)	2.77 ( ± 0.34)	5.2% ( ± 0.6%)	22.69	-2.35

All four spectral bands were used as input.

### 4.2 Impact of spectral information

**Figure 7:**
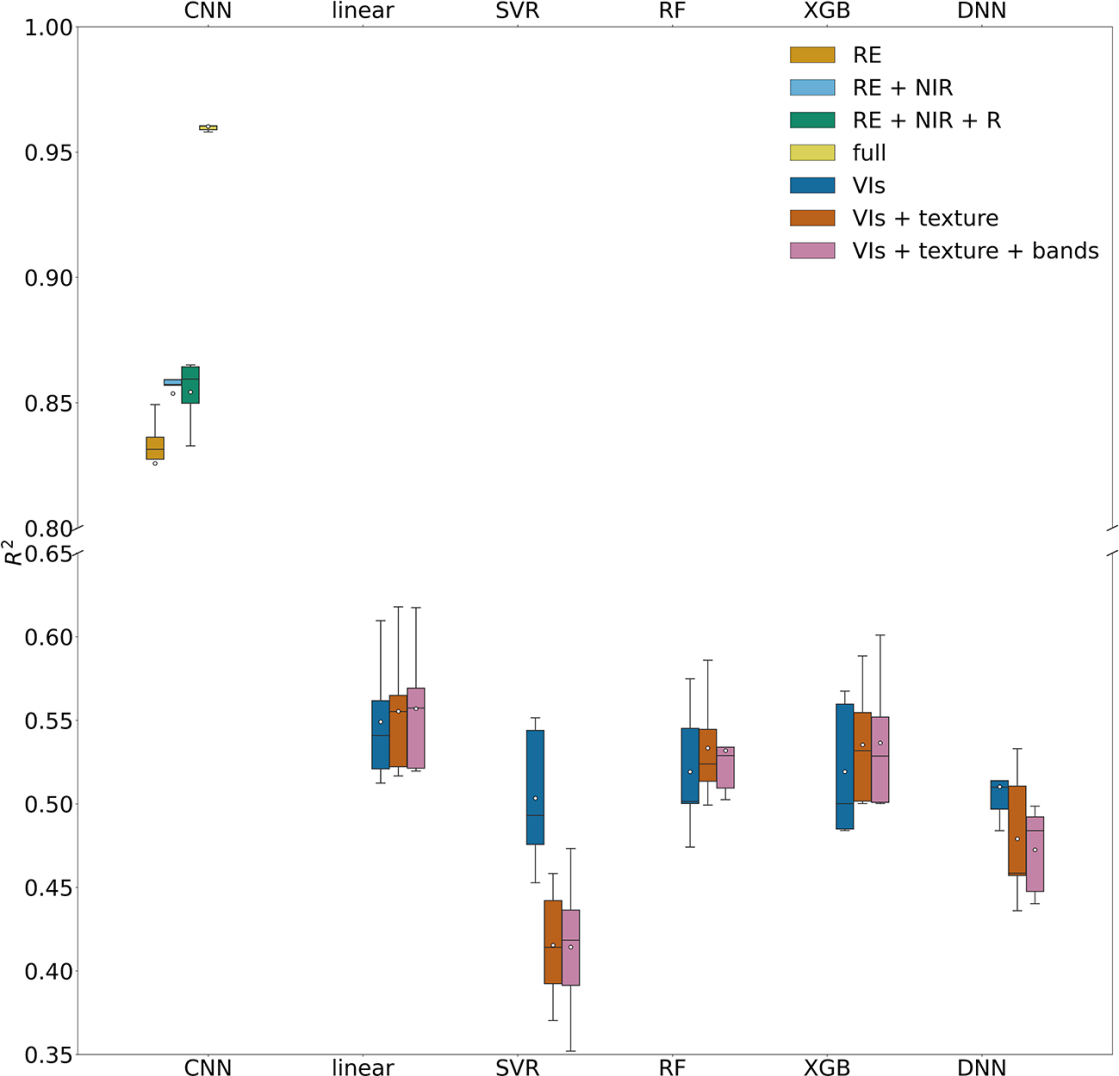
Model performance in predicting tree level yield, quantified by R^2^ with the test data set, for CNN models with different spectral bands and machine learning models with different combinations of input features.

When removing the red edge imagery from the input imagery, the accuracy of the reduced CNN model was reduced significantly, with a lower R^2^ of 0.68 ( ± 0.08) and higher NRMSE of 18.7% ( ± 2.3%) than the full CNN model with four band imagery as input (**Figure 7**). Among the reduced models with all possible combinations of three bands, the CNN model driven by red edge, NIR, and red reflectance performed the best, with a R^2^ of 0.85 ( ± 0.01) and NRMSE of 12.6% ( ± 0.7%). For two band combinations, the reduced model with NIR and red edge bands or NIR and green bands had similar performance (R^2^ 0.85 ( ± 0.02) and 12.6% ( ± 0.8%)). When driven by only one single band imagery, the red edge based CNN model still captured 83% ( ± 2%) of yield variability among individual trees, and NRMSE only increased slightly to 13.8% ( ± 1.0%). These results demonstrated the importance of red edge imagery in almond yield estimation.

### 4.3 Comparison with machine learning models

Our comparison showed that CNN model significantly outperformed the linear regression model and the other machine learning models, based on the 5-fold CV, regardless of combinations of input features such as VIs, texture, and raw multi-spectral reflectance (**Figure 7**). XGB and RF models captured only up to 54% ( ± 3.8%) of yield variability, similar to linear regression models. In addition to achieving the highest R^2^, the CNN model was found more robust and stable as shown by much lower standard deviation of R^2^ among different folds of test sets, compared with other models (**Figure 7**). The scatter plots of predicted vs. measured yield further showed better performance of the CNN model (**Figure 6**).

### 4.4 Predicted yield map and spatial patterns

The CNN full model, once trained and validated, allowed us to estimate yield for every individual almond tree in the orchard. The yield map showed within-field variations of almond yield from tree to tree (**Figure 4A**). Trees with higher yield were mostly located in the northeast corner of the orchard, while least productive trees were mainly distributed around the orchard boundary. The overall spatial pattern was consistent with the pattern captured by the red edge reflectance (**Figure 4B**).

**Figure 8:**
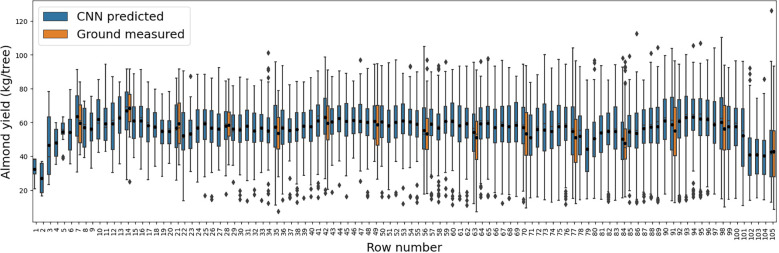
Yield variation within each row as represented by the boxplots of the tree-level yield estimated by the CNN model (blue), and across individual rows. The boxplots of measured yield record for those rows with individual tree harvesting are also shown here in orange for comparison.

When row to row yield variation was examined, the CNN model predicted yield followed similar distribution with the ground measured yields for every seven rows with individual tree yield measurement (**Figure 8**). Row 14 had the highest yield as shown by both estimation (66.9 ± 15.0 kg per tree) and measurements (68.4 ± 13.3 kg per tree); in contrast, the production of Row 84 was 25% lower (50.3 ± 16.1 kg per tree) and 30% lower (47.6 ± 15.4 kg per tree) for both estimation and ground measurements, respectively. The estimation showed large within-row yield variability, with coefficient of variation (CV) ranging from 20.0% to 44.9% and inter-quantile range (IQR) ranging from 16.4 to 31.1 kg per tree, similar to the variability observed by the measurements (**Figure 8**). For rows without ground measurements, the predicted yield also captured similar general trend of row-to-row variation as that from the measurements over the sampled rows.

**Figure 9:**
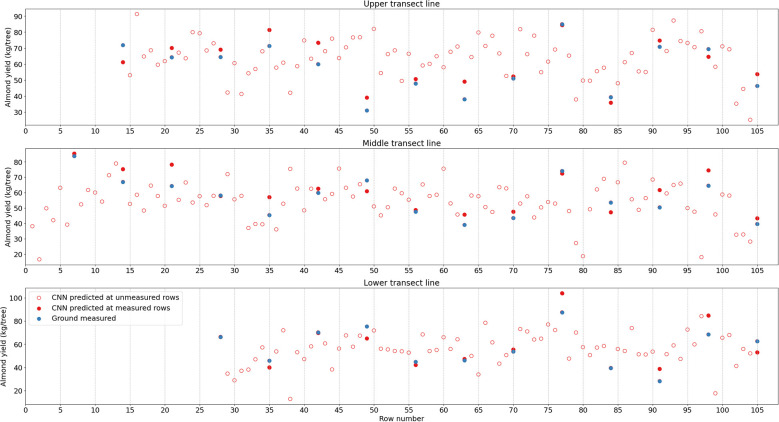
Almond yield variation from tree to tree along three selected transects as shown in **Figure 4**. CNN-estimated yield is represented by red while harvest data in blue; red open circles are for CNN estimation at rows without individual tree yield measurements.

Furthermore, along the transect lines across rows, the inter-row variability from the CNN predicted tree level yield agreed relatively well with that from the ground measurements (**Figure 9**). Among the measured rows, for example, the most productive trees were found in Rows 77 (104.1 kg/tree), 7 (85.4 kg/tree), and 77 (84.4 kg/tree), for each transect, respectively, based on the predicted yield map. In contrast, the least productive trees had much lower yield, i.e., 38.7 kg/tree in Row 91 for transect 1, and 35.9 kg/tree in Row 84 for transect 3. These findings were similar to the observations from the harvesting data. The yield distributions along each row and the inter-row yield variations demonstrated the consistent performance of CNN model over space with less spatial dependency and variations.

## 5 Discussions

### 5.1 Yield estimation model performance

As a first study on tree level almond yield estimation, our findings showed the high accuracy of the CNN model in capturing the spatial yield variability from tree to tree, when driven by multi-spectral reflectance from high resolution aerial imagery. The comparative analysis in this study showed that the CNN model outperforms the traditional machine learning models. First of all, the CNN model framework is able to automatically learn the complex associations from the multi-spectrum tree crown imagery to fully capture the complexity of tree physiology. The spatial pattern of multi-spectral reflectance over the whole crown plays an important role in yield estimation, which cannot be acquired by the average values. For the traditional ML models, the models’ performance generally agrees with literatures using similar features as input for soybean and corn yield estimations. One study focusing on soybean yield estimation with multi-spectrum UAV images shows that models with VIs and thermal information have R2 varying from 0.520 to 0.625 (Maimaitijiang et al., 2020). Based on linear, RF, and XGB results, adding texture features improve model’s ability to explain almond yield variation by 1%, 3%, and 3%, respectively. Some literatures focusing on row crops also have similar finding, but the texture features play a more important role than tree-based plants (Maimaitijiang et al., 2020; Wang et al., 2021). In the soybean study, the VIs, thermal, and structure information explain 52% to 63% of the yield variation with different methods, but adding texture features improves the estimation to explain 65% to 72% of the yield variation, which means that adding the texture features improves about 20% of the estimation accuracy (Maimaitijiang et al., 2020); another rice yield estimation study shows that growing stage VIs explain 56.6% of yield variation and adding extra texture features helps to explain 65.5% yield variation, which increases estimation accuracy by 16% (Wang et al., 2021).

Second, the human-engineered features commonly used by traditional statistical approaches may not fully capture the characteristics influencing yield variation. Most of previous studies focused on crop yield estimation with human-engineered features including VIs and textures, with both ML and AI models showing R^2^s between 0.7 to 0.9 for mostly row crops including wheat, soybean, corn and so on (Kuwata and Shibasaki, 2016; Hunt et al., 2019; Jin et al., 2019; Maimaitijiang et al., 2020; Ma et al., 2021; Wang et al., 2021) and almond orchards at the block level (Zhang et al., 2019). Although these studies use various indices from multi-spectral and thermal UAV images to satellite-based radar backscatter, the estimation accuracy are in general lower than our CNN model with multi-band reflectance as direct inputs. This suggests that human-engineered features may not be comprehensive to fully capture the canopy structures and conditions and yield variations. For example, some information may be lost by only using the well-known remote sensing indices.

Third, super high spatial resolution imagery may improve yield estimation accuracy with more details, especially for deep learning approaches. Gavahi et al. (2021) developed a DeepYield model, which combines convolutional long-short term memory for soybean yield estimation using MODIS Terra and Aqua surface reflectance, land cover type, and surface temperature products. Their results show that the DeepYield model outperforms CNN model with R^2^s of 0.864 over 0.80, which are generally better than many indices-based yield estimation studies. But their yield estimation accuracy is still lower than our CNN model, which is possibly due to their low spatial resolution of input image (500 m and 1 km of MODIS Terra and Aqua products).

### 5.2 Importance of red edge band

From the CNN model result, reflectance in the red edge band was found to play a vital role in almond yield estimation. The red edge spectral band covers a transitional wavelength region from the red band, where the absorption by chlorophyll is dominant, to near infrared where strong scattering by leaf cell structure is further enhanced by multiple scattering among layers of leaves. Reflectance in the red edge band serves as a critical proxy for canopy size and leaf volume. Previous study shows that the red edge band is less saturated at high biomass condition than its adjacent wavelengths and the common vegetation indices such as NDVI (Todd et al., 1998; Mutanga and Skidmore, 2004; Aklilu Tesfaye and Gessesse Awoke, 2021). Moreover, the change in the red edge reflectance may capture some stress conditions of plants, as shown by a recent study on grapevine water stress detection with drone imagery (Tang et al., 2022). Our finding also indicates the potential utility of red edge imagery from Sentinel 2A and 2B satellites for scaling up yield estimation at a large scale.

### 5.3 Uncertainties and future work

This is the first study attempted for the tree-level yield estimation, especially capturing the spatial variability of almond yield within an individual orchard. Although it proves the concept of integrating aerial and drone-based images with deep learning techniques for high resolution yield estimation, some uncertainties still exist. Potential errors, for example, may exist in the harvest yield records used for the model training and testing, as this was the first time the individual tree harvester was designed and tested in the almond field. The sampling strategy, designed by the other group for individual tree harvesting, i.e., every seventh row, prevented us from taking full advantage of the spatial information from neighboring trees for yield estimation in the model building process.

The success of integrating the CNN model with multi-spectral imagery in estimating the within field variability is likely because the imagery at various wavelengths captures the information on the tree structure and plant conditions due to the light-matter interaction. The structural variability such as canopy size can result from cumulative impacts on plant growth by soil properties and long-term climate, while weather variability can also affect the plant health during a particular season. Nonetheless, our study was still constrained by the availability of the yield records for individual trees in one orchard over one single year. Although the unique yield dataset provided sample data covering the gradient of spatial yield variation within a single orchard, it does not represent the yield variability across different orchards where climate and soils may vary significantly. Similarly, the lack of yield record at the tree level from multiple years has prevented us to incorporate weather information in our modeling approach. Future work is needed to collect more ground truthing data and include additional predictors such as soil properties and weather variables for more robust yield estimation and prediction (Zhang et al., 2019).

With rapid advancement in deep learning technology, an important next step is to explore the potential and utility of other powerful approaches such as transformer networks (Vaswani et al., 2017; Liu et al., 2022) and generative adversarial network (Goodfellow Ian et al., 2014). This is particularly helpful for developing a scalable yield estimation workflow, when integrating the time series of high-resolution satellite-based or aerial-based imagery, sometimes at different spatial scales and from different sensors. Remote sensing imagery during the whole growing season and possibly from previous year, for example, can be utilized to integrate the phenological information, e.g., bloom development (Chen et al., 2019), to further improve yield estimation accuracy.

## 6 Conclusion

Individual tree level yield estimation is critical for precision on-farm management and for improving our understanding of yield variability within a field. The challenge of matching efficient supply of inputs like water and fertilizer with tree scale demand is hampered by a lack of understanding of yield variation within orchard blocks. Our work makes a significant step toward bringing awareness to the problem by coupling high-resolution imagery and modeling and paves the way for future innovation in precision orchard management. A CNN deep learning models in estimating almond yield was developed and evaluated, by taking advantage of a unique tree yield data and super high resolution of multi-spectral aerial imagery in 2021 over a single cultivar almond orchard in California’s Central Valley. The 5-fold cross validation showed that the CNN model with spatial attention module, driven by 4-band block imagery of 21 by 21 pixels, captured 96% (±0.2%) of tree-to-tree variation within the study almond orchard with a very low RMSE 3.50 kg/tree and NRMSE of 6.6% ( ± 0.2%). The reduced CNN model with the red edge band reflectance alone had a R^2^ of 0.83 ( ± 0.02) and NRMSE of 13.8% ( ± 1.0%). The CNN model performed significantly better than traditional machine learning methods and stepwise linear regression driven by tree-level features such as VIs and texture.

The almond yield for all individual trees predicted by the CNN model also captured well the spatial patterns and variability of almond yield from row-to-row and from tree-to-tree both within a row and along a transect perpendicular to the row orientation. Our findings demonstrated the potential of applying deep learning technology to integrate high resolution multi-spectral aerial images for accurate and robust tree level yield estimation. The data-driven approach developed here fills an important gap in tree level yield estimation critical for site-specific orchard resource management, ultimately contributing to agriculture sustainability.

## Data availability statement

The original contributions presented in the study are included in the article/**Supplementary Material**. Further inquiries can be directed to the corresponding author.

## Author contributions

MT: conceptualization, methodology, analysis, writing, review and editing. DS, CP, SV, BK, SK, PB: data collection, project coordination, review and editing. YJ: conceptualization, methodology, writing, review and editing. All authors contributed to the article and approved the submitted version.
