# Supplementary material for: Tree-level almond yield estimation from high resolution aerial imagery with convolutional neural network
Source: Front Plant Sci. 2023 Feb 15;14:1070699. doi: 10.3389/fpls.2023.1070699 (PMC9975588; doi:10.3389/fpls.2023.1070699)
Supplement: Supplementary file 1 [file DataSheet_1.docx]

# Tree-level Almond Yield Estimation from High Resolution Aerial Imagery with Convolutional Neural Network

Supplementary material

**1. Individual tree level yield measurement**

We developed a weighing bin to measure individual tree yield. The weighing bin is attached to the commercial almond harvesting machinery as shown in Figure S1.


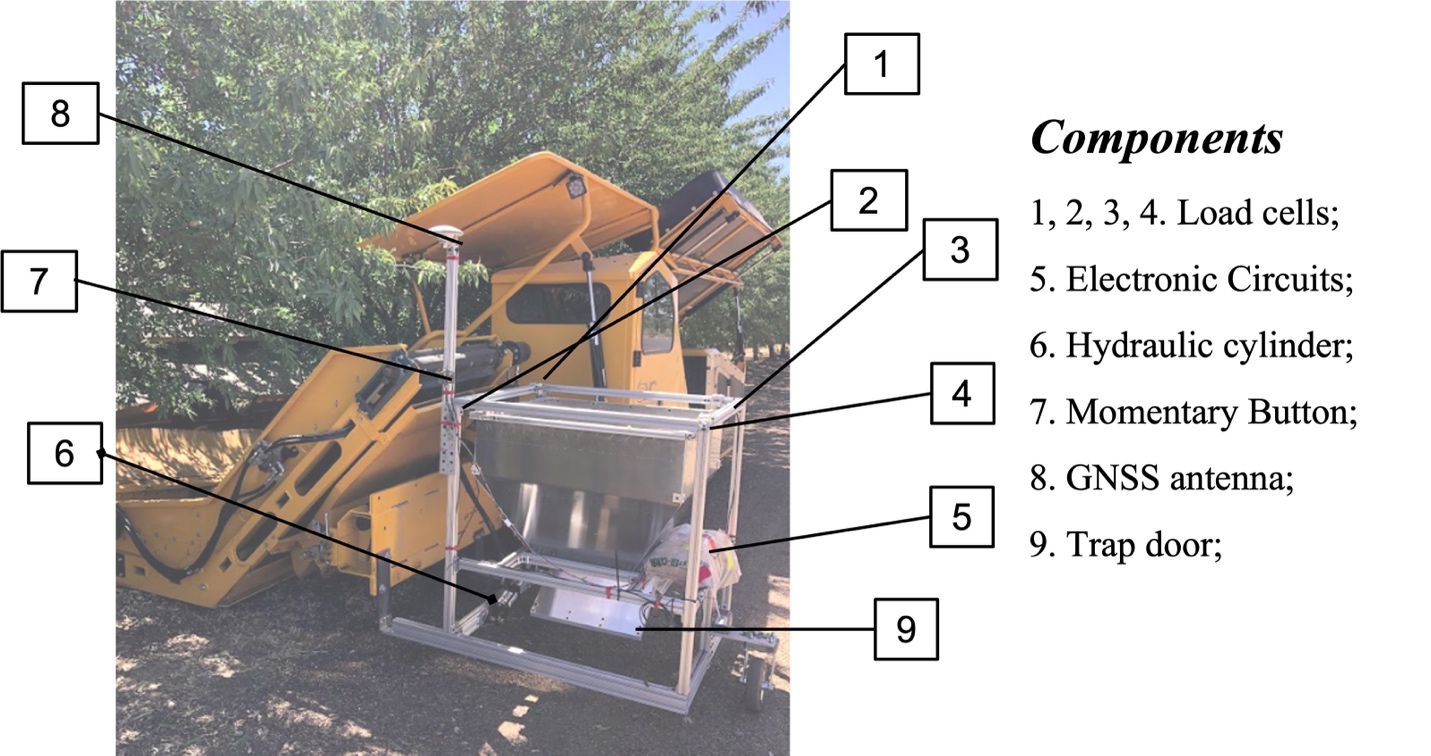


Figure S1. Almond weighting system (lower right) attached to the commercial harvester.

**2. Tree crown segmentation**

To successfully segment each tree crown, we apply the NDVI based multi-threshold and multi-stage segmentation method. We applied 7 NDVI threshold values to segment tree crowns at different level, which is summarized in Table S1. We used the maximum distance within each segmented polygon to decide whether it is a single crown or multiple connected crowns. The detail steps are as follows. First, we produce the pixel-wise NDVI map based on the CERES imagery (Fig. 2a). Second, any pixels with NDVI values greater than the threshold values were identified as tree crown pixels, and the inter-connected tree crown pixels were combined to form the tree crown polygons (Fig. 2b). 7 different layers of tree crown polygon maps were produced according to the 7 different NDVI threshold values.

Third, for each segmented tree crown polygon, the polygons with multiple trees in it were removed from the layer, based on the assumption that one single tree crown diameter can’t exceed the spacing between adjacent trees (or in another word, the maximum possible diameter). For each identified tree crown polygon in Step 2, in order to calculate its dimension in all directions, the length between any two points on the polygon perimeter was calculated and the maximum length was used as the largest crown dimension (similar to “major axis” length of a crown shape) (Fig. 2c). If the major axis of the identified potential tree crown is bigger than the “maximum possible crown diameter” based on the orchard tree planting spacing (~6m), it was flagged as including multiple inter-connected trees and removed from that polygon layer. This process generated seven layers of polygons with only single individual tree crowns.

Finally, segmented single tree crown polygons were combined iteratively based on their spatial relationship to create one final single tree crown polygon map. The goal was to remove the redundancy among those layers yet maintain the largest crown size. Starting from the crown polygons (smallest size), typically associated with higher NDVI threshold value, if it was spatially within the crown polygon (larger) identified by the lower threshold value, the smaller tree crown polygon (identified by the higher threshold values) was deleted and the lower threshold identified polygon was used as a base for next iteration; otherwise, it was added to the final tree crown map. This process was done iteratively to create one final tree crown map, which identified all individual trees within the orchard (solid polygons in Fig. 2d).

For the final tree crown map, a visual check was applied to confirm that all trees have been properly identified and segmented. If there are any missing tree crowns or double tree crown identifications, we will manually fix the issue. Finally, the tree locations were extracted from the centroid coordinates of all the segmented tree crown polygons.

Table S1. NDVI Threshold values for tree crown segmentation.

|  | Threshold 1 | Threshold 2 | Threshold 3 | Threshold 4 | Threshold 5 | Threshold 6 | Threshold 7 |
| --- | --- | --- | --- | --- | --- | --- | --- |
| NDVI value | 0.6 | 0.65 | 0.7 | 0.76 | 0.8 | 0.815 | 0.83 |

**3. CNN model structure optimization**

In this section, we use the Bayesian optimization algorithm to select the optimum CNN structure automatically. The general setup of the possible CNN structures is shown in Figure S2, which includes the range of possible numbers of filters and convolutional block. Within these ranges, Bayesian optimization algorithm will optimize the CNN structure based on prediction accuracy.


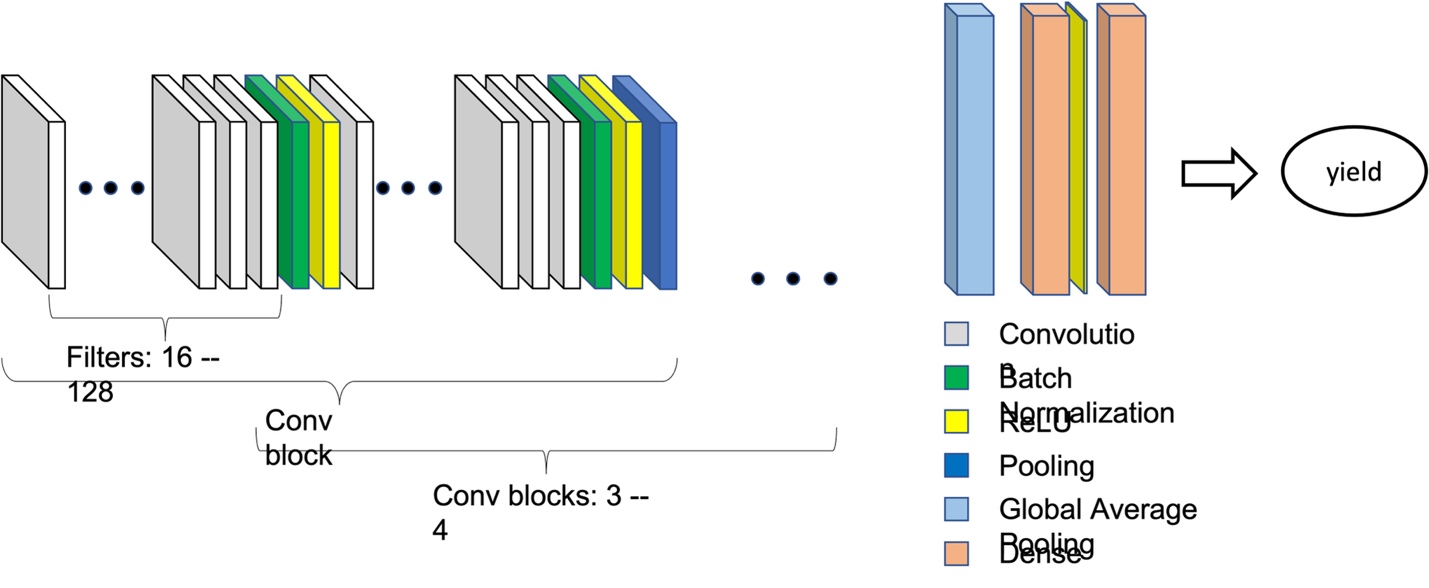


Figure S2. CNN model structures for Bayesian optimization algorithm.

**4. Reduced CNN models**

To compare how the combinations of different reflectance bands influence yield prediction accuracy, we built 14 reduced CNN models with all the possible combinations of different spectral reflectance bands and summarized the performance criteria in Table S2.

Table S2. CNN models’ performance with combinations of different spectral reflectance bands.

| **Bands** | **Test R^2^** | **RMSE** | **NRMSE** |
| --- | --- | --- | --- |
| NIR | 0.59 $(\pm0.04$) | 11.2 (±0.70) | 21.1% (±1.3%) |
| R | 0.63 $(\pm0.04$) | 10.7 (±0.75) | 20.2% (±1.4%) |
| G | 0.15 $(\pm0.29$) | 16.0 (±2.92) | 30.1% (±5.5%) |
| RE | 0.83 ($\pm0.02$) | 7.3 (±0.55) | 13.8% ($\pm1.0\%$) |
| NIR + R | 0.84 ($\pm0.04$) | 6.9 ($\pm0.80$) | 13.0% ($\pm1.5\%$) |
| NIR + G | 0.86 ($\pm0.02$) | 6.6 ($\pm0.34$) | 12.4% ($\pm0.6\%$) |
| NIR + RE | 0.85 ($\pm0.02$) | 6.7 ($\pm0.40$) | 12.6% ($\pm0.8\%$) |
| R + G | 0.81 ($\pm0.03$) | 7.7 ($\pm0.54$) | 14.4% ($\pm1.0\%$) |
| R + RE | 0.80 ($\pm0.04$) | 7.8 ($\pm0.90$) | 14.6% ($\pm1.7\%$) |
| G + RE | 0.81 ($\pm0.02$) | 7.6 ($\pm0.45$) | 14.4% ($\pm0.9\%$) |
| NIR + R + G | 0.68 (±0.08) | 9.9 ($\pm1.23$) | 18.7% (±2.3%) |
| NIR + R + RE | 0.85 (±0.01) | 6.7 ($\pm0.37$) | 12.6% (±0.7%) |
| NIR + G + RE | 0.85 ($\pm0.02$) | 6.8 ($\pm0.53$) | 12.7% ($\pm1.0\%$) |
| R + G + RE | 0.56 ($\pm0.30$) | 11.1 ($\pm3.35$) | 20.9% ($\pm6.3\%$) |
| NIR + R + G + RE (full model) | 0.96 (±0.002) | 3.50 (±0.11) | 6.6% (±0.2%) |

**5. Vegetation Indexes calculated from CERES for traditional machine learning algorithms.**

The VIs can be categorized into three categories: structure indices, greenness indices, and chlorophyll indices (Table S3). For the structure related VIs, NDVI is widely used in plant growth, and vigor related studies (Rouse Jr et al., 1973); soil adjusted vegetation index (SAVI) and optimized soil adjusted vegetation index (OSAVI) are indices developed to reduce the background soil noise (Huete, 1988; Rondeaux et al., 1996). The greenness indices include green band in the calculation, which includes simple ratio green (SRgreen), Green normalized difference vegetation index (GNDVI), and Green-Red Vegetation Index (GRVI) (Chen, 1996; Gitelson & Merzlyak, 1998; Motohka et al., 2010). Chlorophyll indices involves VIs that reflects the canopy chlorophyll content, including Normalized Difference Red Edge (NDRE), Red edge Green Normalized Difference Index (REGI), Canopy Chlorophyll Content Index (CCCI), Chlorophyll Index Green (CIG), Chlorophyll Index Red edge (CIR), and Chlorophyll Vegetation Index (CVI) (Barnes et al., 2000; Datt et al., 2003; El-Shikha et al., 2008; Gitelson et al., 1996, 2006; Klemenjak et al., 2012).

Table S3. Vegetation indices summary.

| Vegetation Index | Abbreviation | Equation | Reference |
| --- | --- | --- | --- |
| ***Structural Indices*** |  |  |  |
| Normalized Difference Vegetation Index | NDVI | $NDVI= \frac{NIR-Red}{NIR+Red}$ | Rouse Jr et al., 1973 |
| Simple Ratio | SR | $SR=\frac{\mathrm{NIR}}{\mathrm{Red}}$ | Chen 1996 |
| Soil-adjusted Vegetation Index | SAVI | $SAVI=1.5\times\frac{NIR-Red}{NIR+Red+0.5}$ | Huete 1988 |
| Optimized Soil-Adjusted Vegetation Index | OSAVI | $OSAVI=\frac{(1+0.16)(NIR-Red)}{NIR+Red+0.16}$ | Rondeaux et al. 1996 |
| ***Greenness Index*** |  |  |  |
| Simple Ratio Green | SR_green_ | $SR.Green=\frac{\mathrm{NIR}}{\mathrm{Green}}$ | Chen 1996 |
| ﻿Green normalized difference vegetation index | GNDVI | $GNDVI=\frac{NIR-Green}{NIR+Green}$ | Mogees et al. 2005 |
| Green-Red Vegetation Index | GRVI | $GRVI= \frac{Green-Red}{Green+Red}$ | Gitelson & Merzlyak, 1998 |
| ***Chlorophyll Indices*** |  |  |  |
| Normalized Difference Red Edge | NDRE | $NDRE=\frac{NIR-Rededge}{NIR+Rededge}$ | Barnese et al. 2020 |
| Rededge Green Normalized Difference Index | REGI | $REGI=\frac{Rededge-Green}{Rededge+Green}$ | Klemenjak et al. 2012 |
| Canopy Chlorophyll Content Index | CCCI | $ccci= \frac{\frac{NIR-Rededge}{NIR+Rededge}}{\frac{NIR-Red}{NIR+Red}}$ | El-Shikha et al., 2008 |
| Chlorophyll Index Green | CIG | $cig= \frac{NIR}{Green}-1$ | Gitelson et al., 1996 |
| Chlorophyll Index Rededge | CIR | $cir= \frac{NIR}{Rededge}-1$ | Gitelson et al., 2006 |
| Chlorophyll Vegetation Index | CVI | $cvi= \frac{NIR\times Red}{{Green}^{2}}$ | Datt et al., 2003 |

**6. Spatial patterns of yield distribution**

We compared the row-wise red edge band reflectance distribution against the row-wise yield distribution to show if there is any clear relation between red edge reflectance and almond yield in Fig. S3. In Fig. S4, the tree-wise red edge band reflectance distribution is compared with the almond tree yield along the three randomly selected transects.


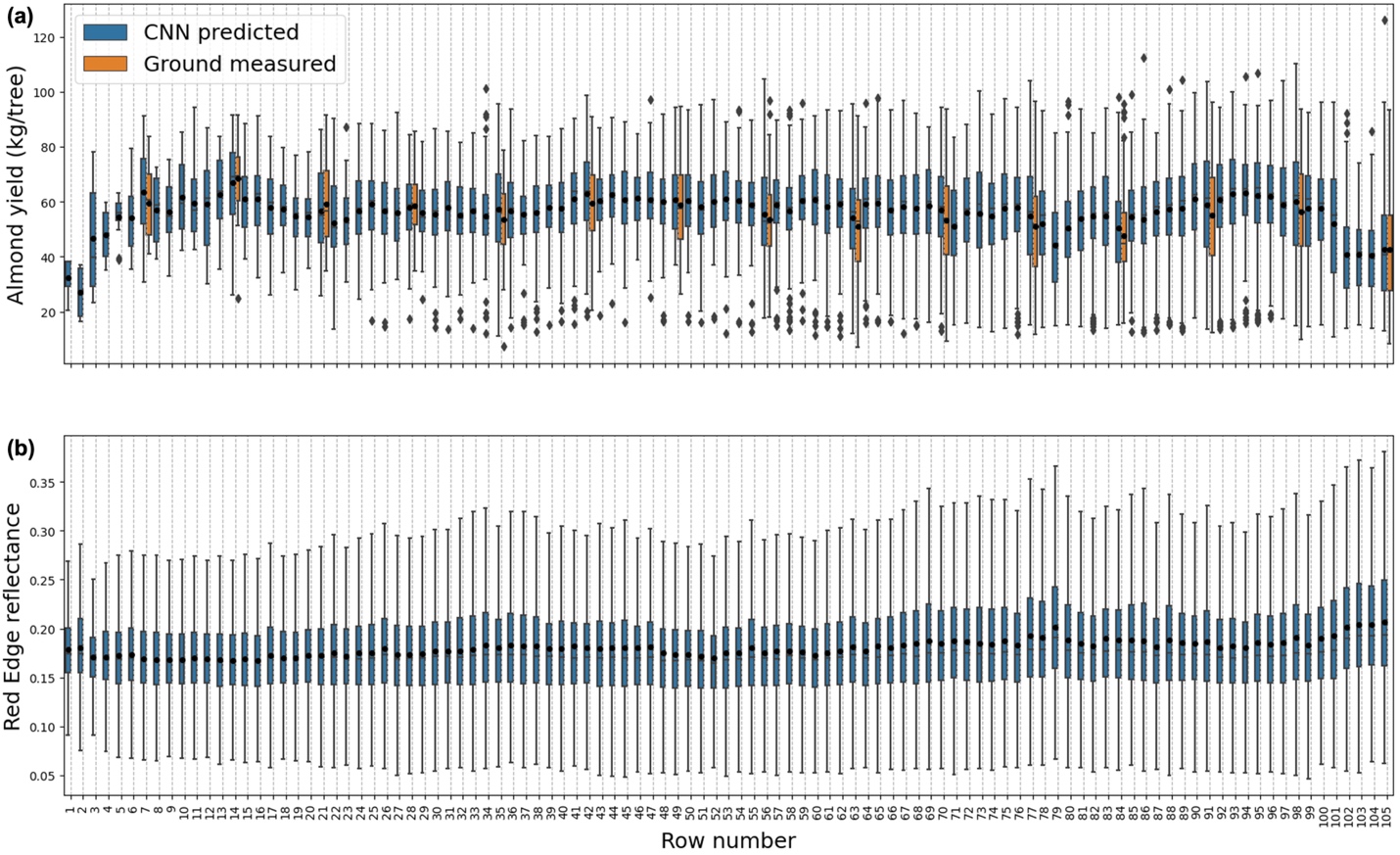


Figure S3. (a) Row-wise almond yield variations and (b) row-wise red edge band reflectance variations.


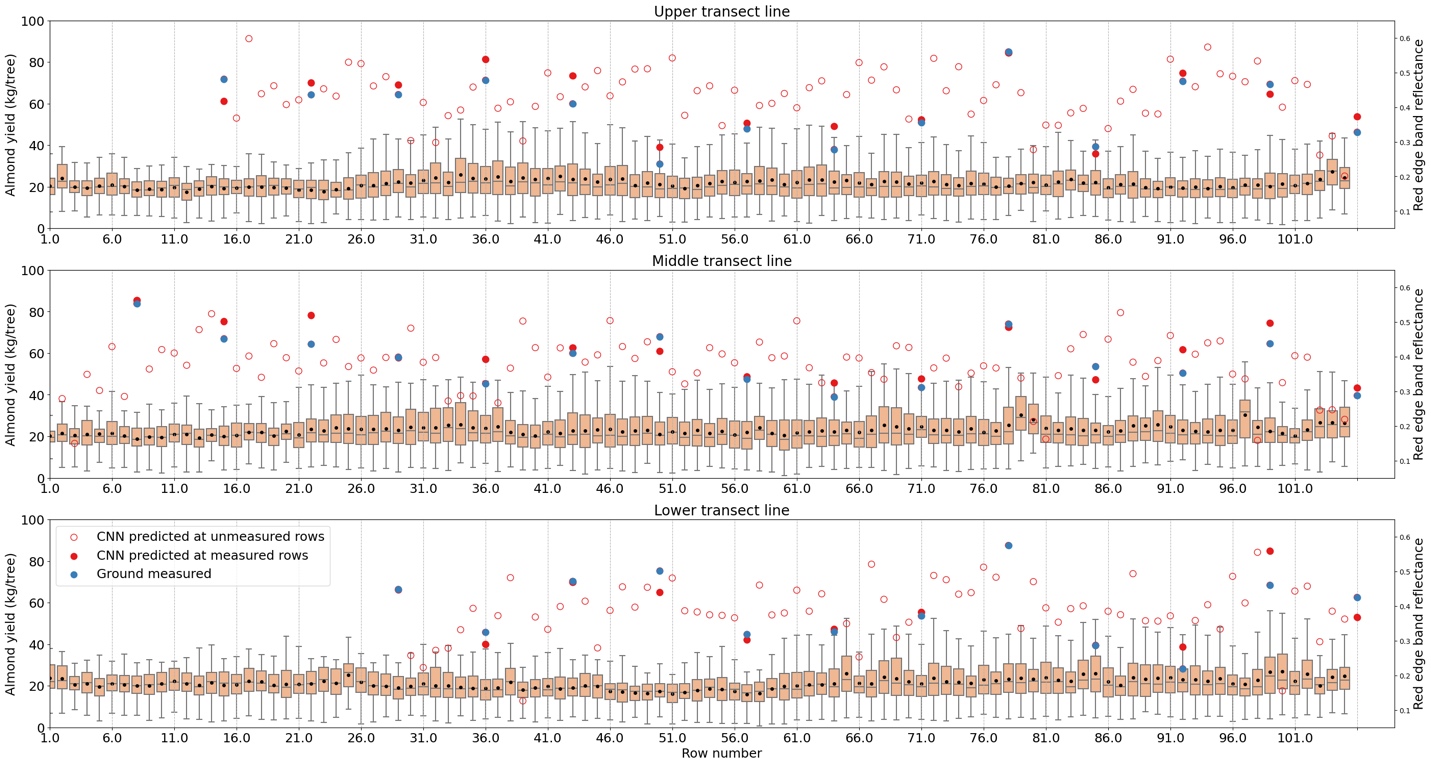


Figure S4. Inter-row almond yield and red edge band reflectance variations. (Red points are predicted yields and blue points are ground measured yields, where red open circle points are predictions at unmeasured rows; boxplots are red edge band reflectance.)

**Reference**

Barnes, E. M., Clarke, T. R., Richards, S. E., Colaizzi, P. D., Haberland, J., Kostrzewski, M., Waller, P., Choi, C., Riley, E., & Thompson, T. (2000). Coincident detection of crop water stress, nitrogen status and canopy density using ground based multispectral data. *Proceedings of the Fifth International Conference on Precision Agriculture, Bloomington, MN, USA*, *1619*.

Chen, J. M. (1996). Evaluation of vegetation indices and a modified simple ratio for boreal applications. *Canadian Journal of Remote Sensing*, *22*(3), 229–242.

Datt, B., McVicar, T. R., Van Niel, T. G., Jupp, D. L. B., & Pearlman, J. S. (2003). Preprocessing EO-1 Hyperion hyperspectral data to support the application of agricultural indexes. *IEEE Transactions on Geoscience and Remote Sensing*, *41*(6), 1246–1259.

El-Shikha, D. M., Barnes, E. M., Clarke, T. R., Hunsaker, D. J., Haberland, J. A., Pinter Jr, P. J., Waller, P. M., & Thompson, T. L. (2008). Remote sensing of cotton nitrogen status using the canopy chlorophyll content index (CCCI). *Transactions of the ASABE*, *51*(1), 73–82.

Gitelson, A. A., Kaufman, Y. J., & Merzlyak, M. N. (1996). Use of a green channel in remote sensing of global vegetation from EOS-MODIS. *Remote Sensing of Environment*, *58*(3), 289–298.

Gitelson, A. A., Keydan, G. P., & Merzlyak, M. N. (2006). Three‐band model for noninvasive estimation of chlorophyll, carotenoids, and anthocyanin contents in higher plant leaves. *Geophysical Research Letters*, *33*(11).

Gitelson, A. A., & Merzlyak, M. N. (1998). Remote sensing of chlorophyll concentration in higher plant leaves. *Advances in Space Research*, *22*(5), 689–692.

Huete, A. R. (1988). A soil-adjusted vegetation index (SAVI). *Remote Sensing of Environment*, *25*(3), 295–309.

Klemenjak, S., Waske, B., Valero, S., & Chanussot, J. (2012). Unsupervised river detection in RapidEye data. *2012 IEEE International Geoscience and Remote Sensing Symposium*, 6860–6863.

Motohka, T., Nasahara, K. N., Oguma, H., & Tsuchida, S. (2010). Applicability of green-red vegetation index for remote sensing of vegetation phenology. *Remote Sensing*, *2*(10), 2369–2387.

Rondeaux, G., Steven, M., & Baret, F. (1996). Optimization of soil-adjusted vegetation indices. *Remote Sensing of Environment*, *55*(2), 95–107.

Rouse Jr, J. W., Haas, R. H., Schell, J. A., & Deering, D. W. (1973). Monitoring Vegetation Systems in the Great Plains with ERTS (Earth Resources Technology Satellite). *Third Earth Resources Technology Satellite-1 Symposium: The Proceedings of a Symposium Held by Goddard Space Flight Center at Washington, DC On*, *351*, 309.
